# Temporal trends of arrhythmias at delivery hospitalizations in the United States: Analysis from the National Inpatient Sample, 2009–2019

**DOI:** 10.3389/fcvm.2022.1000298

**Published:** 2022-11-03

**Authors:** Aarti Thakkar, Yaa A. Kwapong, Harsh Patel, Anum S. Minhas, Arthur J. Vaught, Nicole Gavin, Sammy Zakaria, Roger S. Blumenthal, Katherine C. Wu, Jonathan Chrispin, Sourbha S. Dani, Garima Sharma

**Affiliations:** ^1^Division of Cardiology, Department of Medicine, Ciccarone Center for the Prevention of Cardiovascular Disease, Johns Hopkins University School of Medicine, Baltimore, MD, United States; ^2^Department of Cardiology, Southern Illinois University, Springfield, IL, United States; ^3^Division of Maternal and Fetal Medicine, Department of Gynecology and Obstetrics, Johns Hopkins University School of Medicine, Baltimore, MD, United States; ^4^Division of Maternal Fetal Medicine, Department of Obstetrics and Gynecology, University of Connecticut School of Medicine, Farmington, CT, United States; ^5^Division of Cardiology, Department of Medicine, Johns Hopkins University School of Medicine, Baltimore, MD, United States; ^6^Lahey Hospital & Medical Center, Boston, MA, United States

**Keywords:** trends, arrhythmia, pregnancy, predictors, outcomes

## Abstract

**Background:**

Cardiac arrhythmias are associated with increased maternal morbidity. There are limited data on trends of arrhythmias among women hospitalized for delivery.

**Materials and methods:**

We used the National Inpatient Sample (NIS) database to identify delivery hospitalizations for individuals aged 18–49 years between 2009 to 2019 and utilized coding data from the 9th and 10th editions of the *International Classification of Diseases* to identify supraventricular tachycardias (SVT), atrial fibrillation (AF), atrial flutter, ventricular tachycardia (VT), and ventricular fibrillation (VF). Arrhythmia trends were analyzed by age, race-ethnicity, hospital setting, and hospital geographic regions. Multivariable logistic regression was used to evaluate the association of demographic, clinical, and socioeconomic characteristics with arrhythmias.

**Results:**

Among 41,576,442 delivery hospitalizations, the most common arrhythmia was SVT (53%), followed by AF (31%) and VT (13%). The prevalence of arrhythmia among delivery hospitalizations increased between 2009 and 2019. Age > 35 years and Black race were associated with a higher arrhythmia burden. Factors associated with an increased risk of arrhythmias included valvular disease (OR: 12.77; 95% C1:1.98–13.61), heart failure (OR:7.13; 95% CI: 6.49–7.83), prior myocardial infarction (OR: 5.41, 95% CI: 4.01–7.30), peripheral vascular disease (OR: 3.19, 95% CI: 2.51–4.06), hypertension (OR: 2.18; 95% CI: 2.07–2.28), and obesity (OR 1.69; 95% CI: 1.63–1.76). Delivery hospitalizations complicated by arrhythmias compared with those with no arrhythmias had a higher proportion of all-cause in-hospital mortality (0.95% vs. 0.01%), cardiogenic shock (0.48% vs. 0.00%), preeclampsia (6.96% vs. 3.58%), and preterm labor (2.95% vs. 2.41%) (all *p* < 0.0001).

**Conclusion:**

Pregnant individuals with age > 35 years, obesity, hypertension, valvular heart disease, or severe pulmonary disease are more likely to have an arrhythmia history or an arrhythmia during a delivery hospitalization. Delivery hospitalizations with a history of arrhythmia are more likely to be complicated by all-cause in-hospital mortality, cardiovascular, and adverse pregnancy outcomes (APOs). These data highlight the increased risk associated with pregnancies among individuals with arrhythmias.

## Introduction

Maternal morbidity and mortality have risen steadily in the United States (1–3). In particular, limited data suggest an increased incidence of pregnancy-related arrhythmias, including supraventricular tachycardia (SVT), atrial fibrillation (AF), atrial flutter, and ventricular tachycardia (VT) (4, 5). Although many arrhythmias during pregnancy have been considered benign, there is also limited data suggesting a relationship with in-hospital death. There is also little data associating arrhythmias with comorbidities and risk factors (4). Many cardiovascular risk factors, including preexisting diabetes, obesity, and hypertension increase the risk of adverse pregnancy outcomes (APOs), including preeclampsia and preterm labor, which then contribute to later cardiovascular diseases (6–9). However, the association between arrhythmias before or during delivery and APOs and other cardiovascular outcomes, including cardiogenic shock and cardiac arrest, is poorly understood. It is crucial to recognize comorbidities and risk factors associated with arrhythmias during pregnancy early, particularly if associated with APOs and other cardiovascular outcomes, as it could help guide further risk mitigation (10). The purpose of this paper is threefold: (1) To provide a contemporary analysis of the prevalence of arrhythmias in delivery hospitalizations, (2) To identify outcomes associated with arrhythmias during delivery hospitalizations, and (3) To identify risk factors associated with arrhythmias during delivery hospitalization.

## Materials and methods

### Data source

The study population was derived from the National Inpatient Sample (NIS) dataset (2009–2019), a part of the Healthcare Cost and Utilization Project (HCUP), organized and supported by the Agency for Healthcare Research and Quality (AHRQ) (11). The NIS is the largest all-payer publicly accessible healthcare database in the United States of inpatient encounters. It incorporates a stratified sample of 20% non-federal US community hospitals representing nearly 95% of the US population. It comprises roughly 7 million unweighted records, and about 35 million weighted hospital encounters annually. Weighted data allow us to measure national estimates. The NIS uses de-identified hospital discharges as samples with prior ethical committee approval, therefore, Institutional Review Board (IRB) was not required for this study.

### Study cohort

**FIGURE 1:**
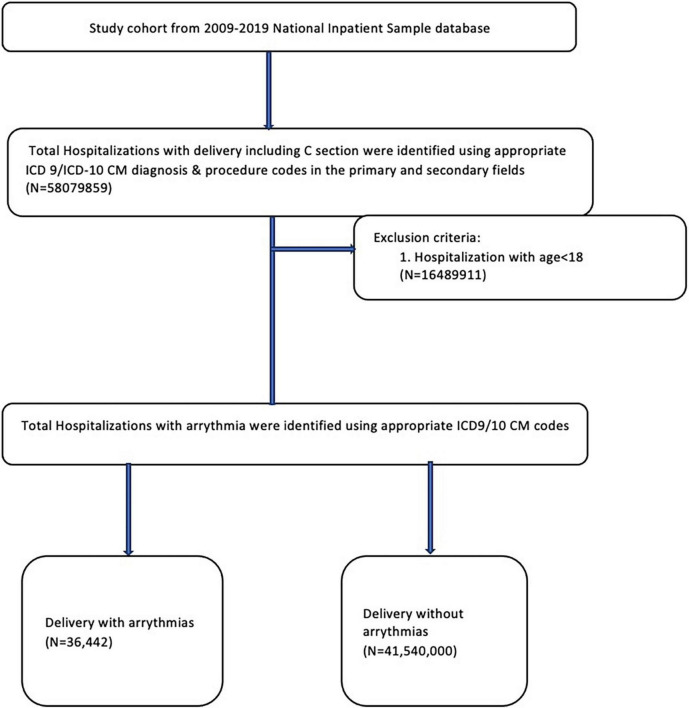
Study population selection algorithm from the National Inpatient Sample database from 2009 to 2019.

We used International Classification of Diseases, 9th and 10th Revision, Clinical Modification (ICD-9 and 10-CM) codes to identify pregnant patients aged 18 years or older hospitalized for delivery. A delivery admission was defined by delivery code (ICD-9 cesarean delivery procedural codes 74 and diagnosis codes 72, 73, 75, v27, or diagnostic codes 650–659 for vaginal delivery; ICD-10 cesarean delivery procedure codes 10D00Z0, 10D00Z1, 10D00Z2, and diagnosis code O82, or vaginal delivery diagnosis codes 060-077, 080, Z37, Z38). Patients were classified into 2 groups based on whether they had prevalent or incident arrhythmia during the same hospitalization (Figure 1). The study cohort was further divided into different age groups (18–24, 25–30, 31–35, 36–40, 41–45, and > 46 years), race/ethnicity groups (White, Black, Hispanic, Asian, Native American, and other), urban or rural settings, and hospital regions (Northeast, Midwest, South, and West). In addition, data on the demographic details, comorbidities, procedure details, and clinical presentation were studied. Specific ICD-9 and ICD-10 codes used to identify comorbidities and outcomes are listed in Supplementary Tables 2, 3.

### Study endpoints

Maternal arrhythmias were characterized according to the various subtypes such as SVT (ICD-9, 427.0; ICD-10, I47.1), AF (ICD-9, 427.31; ICD-10, I48.0, I48.1, I48.2, I48.91), Atrial flutter (ICD-9, 427.32; ICD-10, I48.3, I48.4, I48.92), VT (ICD-9, 427.1; ICD-10, I47.2), and Ventricular fibrillation (VF) (ICD-9, 427.41; ICD-10, I49.01). The primary endpoint was all-cause in-hospital mortality. Secondary endpoints included APOs such as preterm labor, preeclampsia, gestational diabetes, placenta previa; adverse fetal outcomes such as fetal death; mode of delivery such as cesarean section; cerebrovascular outcomes such as ischemic stroke, intracranial hemorrhage; and cardiovascular outcomes such as cardiogenic shock, cardiac arrest, complete heart block and pacemaker implantation, and acute heart failure.

### Data analysis and statistics

As recommended, survey procedures using discharge weights provided with the HCUP-NIS database were used to generate national estimates. Descriptive statistics were used to analyze the demographic and comorbidity data. Most hospital-level characteristics were directly obtained as provided in the NIS, whereas the Elixhauser Comorbidity Index was used to identify co-morbid disorders (12). Categorical variables were compared using the Chi-square test and are presented as numbers and percentages. Continuous variables were compared using the Wilcoxon test and are presented as the median and interquartile range (IQR). A two-tailed *p*-value of < 0.05 was used to determine the statistical significance. We performed multivariable logistic regression to look for risk factors associated with arrhythmia in delivery hospitalization. Furthermore, the Jonckheere-Terpstra trend test was used to analyze various trends, including the overall frequency of arrhythmia, frequency of arrhythmia stratified by age group, ethnicity, hospital setting, and hospital geographic regions in delivery hospitalizations from 2009 to 2019. All analyses were performed using SAS, version 9.4 (SAS Institute Inc.).

## Results

### Overall delivery hospitalizations with arrhythmia

**FIGURE 2:**
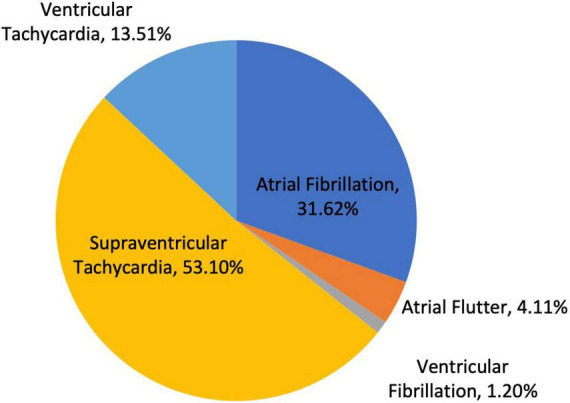
Frequency of arrhythmia subtypes in all delivery hospitalizations; specifically atrial fibrillation, atrial flutter, supraventricular tachycardia, ventricular tachycardia, and ventricular fibrillation.

During the study period from 2009 to 2019, there were a total of 41,576,442 delivery hospitalizations. Among delivery hospitalizations, there were 36,442 hospitalizations associated with any diagnosis of arrhythmia either during or prior to the delivery hospitalizations. The most common arrhythmia diagnosis was SVT (53%), followed by AF (31%) and VT (13%) (Figure 2).

### Demographic data

The baseline demographics of delivery hospitalizations with and without an arrhythmia are shown in Table 1. Delivery hospitalizations with an arrhythmia were more likely in older age groups than those without an arrhythmia (31–35 years: 26.58% vs. 23.71; 36–40 years: 15.15% vs. 10.86%; 41–45years: 4.27% vs. 2.07%, all *p* < 0.0001). There were also significant differences in race/ethnicity in delivery hospitalizations with an arrhythmia compared with those without. (White: 60.13% vs. 52.82%; Black: 20.44% vs. 14.77%; Hispanic: 11.35% vs. 21.13%; Asian: 3.94% vs. 5.76, all *p* < 0.0001). Additionally, there were rural and urban differences in delivery hospitalizations associated with arrhythmias, where 73.66% of delivery hospitalizations with an arrhythmia occurred in urban teaching hospitals. In comparison, 59.29% without arrhythmia occurred in urban teaching hospitals (*p* < 0.0001). In addition, delivery hospitalizations with arrhythmia had a higher proportion of private insurance status (54.89% vs. 50.79%, *p* < 0.001) compared with those with no arrhythmia.

**TABLE 1:** Demographics and baseline characteristics of delivery hospitalizations.

	Arrhythmia (*N* = 36,442)	No arrhythmia (*N* = 41,540,000)	*P*-value
**Age groups**			<0.0001
18–24	17.25%	25.10%	
25–30	36.74%	38.26%	
31–35	26.58%	23.71%	
36–40	15.15%	10.86%	
41–45	4.27%	2.07%	
>46	−	−	
**Race**			<0.0001
White	60.13%	52.82%	
Black	20.44%	14.77%	
Hispanic	11.35%	21.13%	
Asians	3.94%	5.76%	
Native Americans	0.65%	0.79%	
Others	3.50%	4.74%	
**Type of admission**			<0.0001
Elective	45.18%	49.39%	
Non-elective	54.82%	50.61%	
**Primary payer**			<0.0001
Medicare	2.32%	0.71%	
Medicaid	37.58%	42.81%	
Private insurance	54.89%	50.69%	
Other/Self-pay	5.21%	5.79%	
**Median income (quartile)**			0.0002
0–25th percentile	26.69%	27.61%	
26–50th percentile	24.94%	25.02%	
51–75th percentile	25.33%	25.00%	
76–100th percentile	23.04%	22.37%	
**Hospital bedside**			<0.0001
Small	11.90%	14.81%	
Medium	24.90%	29.05%	
Large	63.20%	56.14%	
**Hospital region**			<0.0001
Northeast	16.97%	16.06%	
Midwest	21.14%	21.14%	
South	38.25%	38.54%	
West	24.25%	24.26%	
**Hospital location and teaching status**			<0.0001
Rural	6.97%	9.99%	
Urban non-teaching	19.37%	30.72%	
Urban teaching	73.66%	59.29%	

Comparisons were made by chi-squared for categorical variables. Statistical significance set at *p* < 0.05; SD, Standard deviation.

### Comorbidities

The clinical comorbidities and complications of delivery hospitalizations with and without arrhythmia are detailed in Table 2. Individuals with delivery hospitalizations with an arrhythmia were more likely to have cardiovascular risk factors including obesity (16.95% vs. 8.31%), hypertension (6.19% vs. 1.53%), type II diabetes (1.39% vs. 0.64%), previous myocardial infarction (0.22% vs. 0.01%), peripheral vascular disease (0.23% vs. 0.01%), and hyperlipidemia (0.35% vs. 0.06%) compared with those without arrhythmias (all *p* < 0.001). Individuals with arrhythmias, compared with those without, had a higher prevalence of risk behaviors such as tobacco use (2.15% vs. 1.36%) and other substance abuse (2.17% vs. 1.38%) (all *p* < 0.0001). Additionally, delivery hospitalizations were more likely to have comorbidities such as heart failure (3.02% vs. 0.04%), valvular disease (4.09% vs. 0.13%), chronic obstructive pulmonary disease (7.41% vs. 2.84%), fluid and electrolyte disorders (5.03% vs. 0.34%), hypothyroidism (4.02% vs. 2.06%), and coagulopathy (4.14% vs. 1.37%) as compared to those who did not have any arrhythmias (all *p* < 0.0001).

**TABLE 2:** Comorbidities and complications of women with and without arrhythmia at delivery hospitalization.

	Arrhythmia (*n* = 36,442)	No arrhythmia (*n* = 41,540,000)	*P*-value
**CV risk factors**			
Obesity	16.95%	8.31%	<0.0001
Hypertension	6.91%	1.54%	<0.0001
Hyperlipidemia	0.35%	0.06%	<0.0001
Type 2 DM	1.39%	0.64%	<0.0001
Previous MI	0.22%	0.01%	<0.0001
Peripheral vascular disease	0.23%	0.01%	<0.0001
**Risk behaviors**			
Tobacco use	2.15%	1.36%	<0.0001
Alcohol abuse	0.10%	0.07%	0.04
Substance abuse	2.17%	1.38%	<0.0001
**CV comorbidities**			
Heart failure	3.02%	0.04%	<0.0001
Valvular heart disease	4.09%	0.13%	<0.0001
**Extracardiac comorbidities**			
COPD	7.41%	2.84%	<0.0001
Renal failure	0.44%	0.05%	<0.0001
Hypothyroidism	4.02%	2.06%	<0.0001
Fluid and electrolyte disorders	5.03%	0.36%	<0.0001
Coagulopathy	4.14%	1.37%	<0.0001
Pulmonary circulation disease	0.58%	0.01%	<0.0001

Comparisons were made by ANOVA for continuous variables and a chi-squared for categorical variables. Statistical significance set at *p* < 0.05. COPD, chronic obstructive pulmonary disease; CV, cardiovascular; DM, diabetes mellitus; MI, myocardial infarction.

### Temporal trends in prevalence and baseline characteristics of hospitalizations with arrhythmia

**FIGURE 3:**
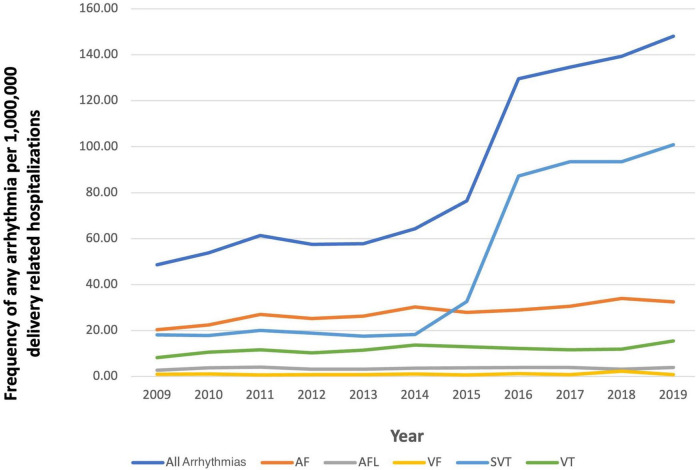
Frequency of any arrhythmia per 1,000,000 delivery related hospitalizations from 2009 to 2019, stratified by type of arrhythmia.

**FIGURE 4:**
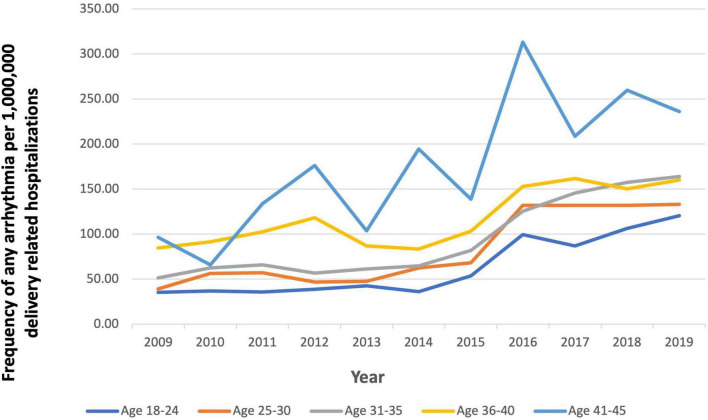
Frequency of any arrhythmia per 1,000,000 delivery related hospitalizations from 2009 to 2019, stratified by age.

**FIGURE 5:**
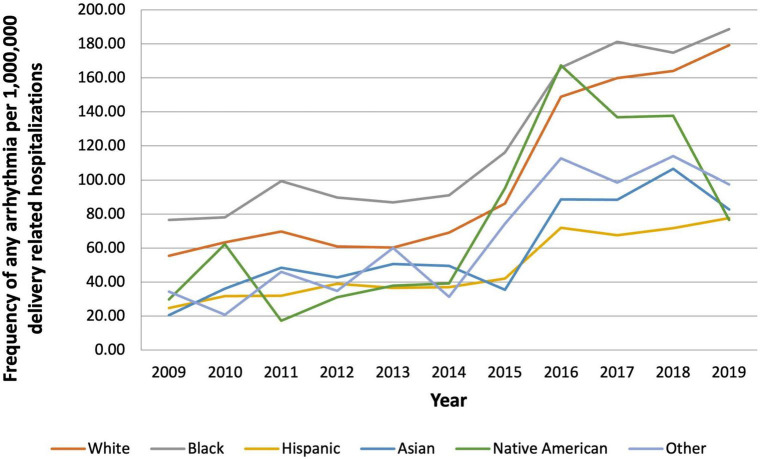
Frequency of any arrhythmia per 1,000,000 delivery related hospitalizations from 2009 to 2019, stratified by race.

**FIGURE 6:**
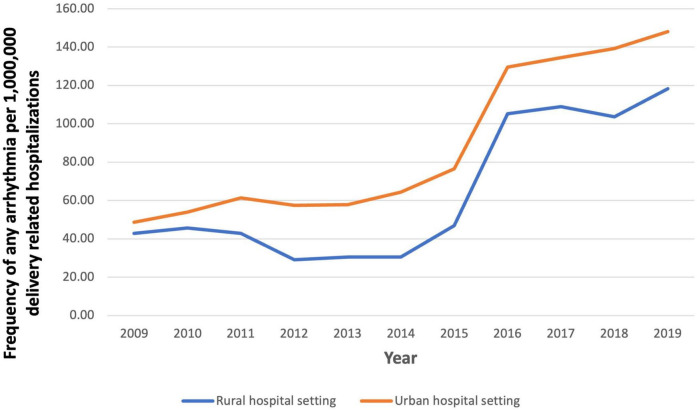
Frequency of any arrhythmia per 1,000,000 delivery related hospitalizations from 2009 to 2019, stratified by hospital setting.

In 2009, the prevalence of arrhythmias was 48.67 per 1,000,000 in delivery hospitalizations, which increased to 148.17 per 1,000,000 (Figure 3). From 2009 to 2019, SVT increased the most from 18.13 to 100.84 per 1,000,000 delivery hospitalizations. Patients in the oldest age groups (36–40 and 41–45) had the highest prevalence of an arrhythmia compared to women in younger age groups (18–24, 25–30, and 31–35) (Figure 4). For almost every year, Black women had the highest prevalence of arrhythmia per 1,000,000 delivery hospitalizations compared to other races (Figure 5). Hospitals in urban settings had a higher prevalence of arrhythmia per 1,000,000 delivery hospitalizations across the entire study period compared to hospitals in rural settings (Figure 6).

### Outcomes of patients with arrhythmia

Delivery hospitalizations in women with a history of arrhythmia had a significantly higher proportion of all-cause in-hospital mortality (0.95% vs. 0.01%, *p* < 0.0001) as well as other poor clinical outcomes (Table 3). There was also a higher prevalence of various APOs: preeclampsia (6.96% vs. 3.58%), preterm labor (2.95% vs. 2.41%), placental abruption (0.67% vs. 0.26%), placenta previa (0.33% vs. 0.11%) (all *p* < 0.001) and gestational diabetes mellitus (3.69% vs. 1.74%; *p* = 0.04) compared with those with no arrhythmia. The delivery hospitalizations with a history of arrhythmia were more associated with cesarean section (43.48% vs. 31.97%), fetal death (0.21% vs. 0.04%), cardiac arrest (1.32% vs. 0.00%), and cardiogenic shock (0.48% vs. 0.0%) (*p* < 0.0001).

**TABLE 3:** Outcomes in delivery hospitalizations with and without arrhythmias.

	Arrhythmia (*n* = 36,442)	No arrhythmia (*n* = 41,540,000)	*P*-value
**All-cause in-hospital mortality**	0.95%	0.01%	<0.0001
**Adverse pregnancy outcomes**			
Gestational diabetes mellitus	3.69%	1.74%	0.04
Preterm labor	2.95%	2.41%	<0.0001
Preeclampsia	6.96%	3.58%	<0.0001
Placental abruption	0.67%	0.26%	<0.0001
Placenta previa	0.33%	0.11%	<0.0001
Fetal death	0.21%	0.04%	<0.0001
**Obstetrics outcomes**			
Cesarean section	43.48%	31.97%	<0.0001
**CV outcomes**			
Ischemic stroke	0.08%	0.00%	<0.0001
Cardiogenic shock	0.48%	0.00%	<0.0001
Cardiac arrest	1.32%	0.00%	<0.0001
Acute heart failure	1.55%	0.01%	<0.0001
**Other**			
ICH	0.05%	0.00%	<0.0001

Comparisons were made by ANOVA for continuous variables and a chi-squared for categorical variables. Statistical significance set at *p* < 0.05. OR, Odds Ratio; CI, Confidence Interval; CV, Cardiovascular; CM, Cardiomyopathy; ICH, Intracranial hemorrhage.

### Factors associated with delivery hospitalizations complicated with arrhythmias

Multivariable logistic regression for the presence of arrhythmia during delivery hospitalization is shown in Table 4. Inspecting demographic predictors of arrhythmias showed that per each 1-year increase in age, the odds of an arrhythmia increased by 4% (OR: 1.04; 95% CI: 1.03–1.04). When controlled for other risk factors, Black women had 2.05 times greater odds (95% CI: 1.95–2.12) of an arrhythmia than Hispanic women, and White women had 1.91 times greater odds (95% CI: 1.82–1.99) of an arrhythmia than Hispanic women.

**TABLE 4:** Comparisons were made by ANOVA for continuous variables and a chi-squared for categorical variables.

	OR [95% CI]
Age	1.04 [1.03–1.04]
**Type of admission**	
Elective	0.86 [0.83–0.88]
**Race**	
Hispanic	Reference
Black	2.05 [1.95–2.15]
White	1.91 [1.83–1.99]
Asian	1.15 [1.07–1.24]
Native American	1.60 [1.36–1.87]
Others	1.37 [1.27–1.48]
**Payee status**	
Self-pay/other	Reference
Medicare	1.51 [1.42–1.61]
Medicaid	0.96 [0.93–1.00]
Private insurance	0.98 [0.95–1.01]
**Median household income (percentile)**	
0–25th	Reference
25th–50th	1.05 [1.01–1.09]
50th-75th	1.02 [0.98–1.06]
75th–100	1.02 [0.98–1.07]
**Comorbidities**	
Obesity	1.69 [1.63–1.76]
Hypertension	2.18 [2.07–2.28]
Hyperlipidemia	1.50 [1.20–1.87]
Diabetes mellitus	0.92 [0.84–1.02]
Hypothyroidism	1.50 [1.42–1.58]
CHF	7.13 [6.49–7.83]
Chronic pulmonary disease	1.92 [1.84–2.00]
Renal failure	1.26 [1.05–1.50]
Pulmonary embolus	1.55 [1.30–1.85]
Tobacco use	1.12 [1.03–1.23]
Alcohol abuse	0.75 [0.53–1.05]
Drug abuse	1.07 [0.99–1.16]
Valvular disease	12.77 [11.98–13.61]
Fluid and electrolyte disorders	6.57 [6.21–6.96]
Coagulopathy	1.94 [1.83–2.05]
Peripheral vascular disease	3.19 [2.51–4.06]
Old myocardial infarction	5.41 [4.01–7.30]

Statistical significance set at *p* < 0.05. CHF, Heart failure; OR, Odds Ratio; CI, Confidence Interval; ICH, intracranial hemorrhage.

Cardiovascular comorbidities, including valvular disease and heart failure, significantly increased the odds of an arrhythmia during pregnancy by 12.77 (95% CI: 11.98–13.61) and 7.13 (95% CI 6.49 –7.83), respectively. In addition, the presence of cardiovascular risk factors, including obesity (OR: 1.69; 95% CI: 1.63–1.76), hypertension (OR: 2.18; 95% CI: 2.07–2.28), previous MI (OR: 5.41, 95% CI: 4.01–7.30), and peripheral vascular disease (OR: 3.19, 95% CI: 2.51–4.06), also increased the odds of reported arrhythmias though to a slightly lesser degree.

Regarding socioeconomic factors, women with Medicare status were 1.5 times more likely to have arrhythmias (OR 1.51, 95% CI 1.423–1.61) than those who self-pay. There was no association of arrhythmia with median household income or substance abuse.

## Discussion

In this large nationally represented sample of delivery hospitalizations from 2009 to 2019, we report the overall prevalence and incidence and temporal trends of arrhythmia frequency in pregnancy-related hospitalizations. Our major conclusions from this analysis show: (1) the overall combined prevalence and incidence of arrhythmias increased by ∼200% from 48.67/1,000,000 to 148.14/1,000,000 from 2009 to 2019, (2) there was an increased APO frequency, cardiovascular and obstetric complications, and slightly higher in-hospital mortality, among women with preexisting or incident arrhythmias, (3) there was a higher association of arrhythmias with older age, non-Hispanic race, and certain cardiovascular conditions and obstetric conditions.

These findings build upon previously published studies, which have all shown an increased frequency of arrhythmias in pregnant women since the 1990s (4, 5). Our findings demonstrate a similar increase in arrhythmias from 2009 to 2019. The measured rise in prevalence of arrhythmias during pregnancy is likely multifactorial. While the increased prevalence could be partially the result of a rise in risk factors, such an increase could be also be due to greater use of electronic medical records, remote monitoring, and overall shifts in monitoring and recording practices. Additionally, many health systems shifted from ICD-9 and ICD-10 over the examined time frame which may have also contributed to a shift in identification and coding thus leading to a perceived increase in arrhythmias from 2009 to 2019. In another report of pregnancy hospitalizations from 2000 to 2012 AF was the most frequent arrhythmia in pregnancy; however, our results show that SVT is more common, which is also the most significant contributor to the rise in overall arrhythmias from 2009 to 2019. Our findings are most similar to a cohort of pregnant women with congenital heart disease, where SVT was also the most commonly reported arrhythmia (13).

This study highlights that presence of any arrhythmia is associated with a slightly greater risk of in-hospital mortality among women hospitalized for delivery. Arrhythmias were also associated with increased obstetric complications, including increased likelihood of Cesarean section, cardiovascular complications, including cardiac arrest, and APOs, specifically preeclampsia and preterm labor. Such adverse outcomes have their own health consequences and are not self-limited, leading to increased risk for short-term and long-term maternal morbidity (14–17). This analysis was unable to elucidate if one specific arrhythmia was more associated with in-hospital mortality or specific negative outcomes. However, further studies examining contribution of SVT, which is thought to be more benign, in comparison to VT, VF, or AF, with regards to mortality would provide great clinical significance for providers as they attempt to risk stratify their patients in real time. As maternal morbidity rises globally, early recognition of risk factors becomes more crucial to preventing APOs and downstream complications.(1, 18, 19) At the very least, the presence of an arrhythmia might help identify a higher risk group that will benefit from addressing cardiovascular health during pregnancy and in the post-partum time frame.

Since arrhythmias are markers for poor maternal outcomes, identifying sociodemographic and clinical risk factors that increase arrhythmia risk are crucial for risk mitigation. Our analysis shows that older pregnant individuals hospitalized for delivery, particularly those of advanced maternal age (age > 35), had a higher frequency of prevalent and incident arrhythmia. This is not unexpected, as aging in the general population has been associated with increased prevalence and severity of arrhythmias.(20) Also, increased maternal age is associated with cardiovascular comorbidities such as diabetes, hypertension, dyslipidemia, and obesity, which may partly contribute to the increased risk of arrhythmia. As the mean age of mothers continues to rise, it is essential to recognize the increased risk of arrhythmias in this population when counseling before and during pregnancy (21). Notably, our analysis was primarily focused on acquired risk factors. The specific contribution of having a history of congenital heart disease or inherited arrhythmogenic conditions—both of which have known correlation with increased risk of arrhythmia—were not elucidated in this study (22, 23). However, the considerable contribution of such history should not be forgotten and must be included in clinical practice when working with pregnant populations.

Beyond demographics, general cardiovascular risk factors such as obesity, hypertension, and known coronary artery disease with prior myocardial infarction further increased risk a woman’s risk for arrhythmia among delivery hospitalizations. Individuals who are contemplating pregnancy or are pregnant with these risk factors should undergo aggressive risk factor modification and lifestyle modification. Our data also emphasize that those with known history of structural cardiac disease have an increased risk for arrhythmia and maternal morbidity—with a seven times greater risk for those with heart failure and 12 times greater risk for those with valvular disease. In addition, there are important differences in risk depending on race and ethnic status. Overall arrhythmias are highest in White pregnant individuals compared to other races; however, Black individuals had the highest yearly prevalence of arrhythmia. During the study time period, the prevalence of arrhythmia increased greatly for both Black and White pregnant individuals from 76.59/100,000 to 188.70/100,000 for Black (a 146% increase) and 55.49/100,000 to 179.19/100,000 for White (a 225% increase). Our results are in line with other NIS studies which have shown that Black pregnant individuals face a disproportionate burden of preeclampsia, peripartum cardiomyopathy, and severe maternal morbidity (24–26).

Socioeconomic factors may also be associated with differences in arrhythmia prevalence. Individuals on Medicare are at increased risk of arrhythmia compared to those with private insurance. Since almost all patients in their peripartum years are younger than the typical year of eligibility for Medicare, it is likely that these patients have renal failure, end-stage renal failure, or other serious that qualify them for this coverage antenatally. In contrast, there is no association between arrhythmias and median household income. While other studies evaluating members of the general population reveal a lower risk of AF for those with a higher socioeconomic status than their age and race-matched counterparts (27), our study did not demonstrate the same trends. However, our population is different, as it includes only women who were hospitalized for delivery, who are younger than other study populations. Thus, the cascading downstream impact of social determinants of health may not yet have manifested clinically.

All women with known CVD or at increased CVD risk benefit from multidisciplinary care and preventive cardiology intervention prior to pregnancy and antenatally. As more individuals with simple or complex cardiovascular history are becoming pregnant, interdisciplinary cardio-obstetric collaboration has been shown to reduce cardiac maternal morbidity.(10, 28–30) Management of arrhythmia must occur at every stage of pregnancy, from prevention to early recognition of complications during delivery. Current risk scores for cardiovascular maternal morbidity, such as ZAHARA (Zwangerschap bij vrouwen met een Aange- boren HARtAfwijking-II) and CARPREG II (Cardiac Disease in Pregnancy II) already include history of prior cardiac events or arrhythmias in their score (31–33). Implementing interventions on cardiovascular comorbidities of pregnant women are necessary to reduce the growing morbidity and mortality of arrhythmia.

### Study limitations

As with other studies which utilize the NIS, our study does face several limitations. First, in this deidentified NIS sample, we are unable to differentiate between pregnant individuals who may have had multiple admissions during the study period. Second, large databases are at risk of errors in coding, and we are unable to validate arrhythmia categorization, electrocardiogram analysis, causes of death, pregnancy outcomes, and long-term-follow up and other arrhythmia monitoring practices. Third, the database is not able to differentiate between new arrhythmias during the delivery hospitalization vs. a prior history of arrhythmia earlier in the patient’s life. Fourth, we were unable to fully characterize patients with multiple admissions prior to the delivery. Fifth, we are unable to determine long-term outcomes of delivery hospitalizations complicated with arrhythmias due to database limitations. Finally, the NIS database did not include all characteristics that we would hope to examine including, but not limited to, an analysis of outcomes from hospitals that offer high-risk obstetric care verses those that do not, parity of delivery, and outcomes from natural pregnancies verses those that occurred with the use of assisted reproductive therapies. Furthermore, this analysis does not specifically evaluate the contribution of congenital heart disease or inherited arrhythmia conditions, such as arrhythmogenic cardiomyopathy or catecholaminergic polymorphic VT, to arrhythmia during pregnancy. Despite these limitations, our study is one of the largest evaluating delivery hospitalizations associated with arrhythmias from data across the United States providing greater generalizability.

## Conclusion

In conclusion, we note an increasing combined prevalence and incidence of arrhythmias among women hospitalized for pregnancy in recent years. Pregnant individuals with underlying comorbidities, such as obesity, hypertension, structural heart disease, and heart failure, are more likely to be associated with tachyarrhythmias. Additionally, those hospitalized for delivery with arrhythmias prior to or during their pregnancy are more likely to have APOs, such a preeclampsia, preterm birth, and cesarean sections. This study allows physicians and patients to recognize recent trends in arrhythmias and related complications for pregnant women.

## Data availability statement

Publicly available datasets were analyzed in this study. This data can be found here: National Inpatient Sample Database, https://www.hcup-us.ahrq.gov/db/nation/nis/nisdbdocumentation.jsp.

## Author contributions

AT substantially contributed to the conception and design of the work, interpreted the results, drafted the first version of the manuscript, substantially revised the manuscript, and approved the final manuscript. YK, HP, SD, and GS substantially contributed to the conception and design of the work, acquisition and analysis of the data, verification of the underlying data, interpretation of the results, revised the manuscript, and approved the final manuscript. AM, AV, NG, SZ, RB, KW, and JC substantially contributed to the conception and design of the work, interpreted the results, revised the manuscript, and approved the final manuscript. All authors contributed to the article and approved the submitted version.

## Abbreviations

AF: Atrial fibrillation
APO: Adverse Pregnancy Outcomes
CVD: Cardiovascular Disease
ICD: International Classification of Diseases
IQR: Interquartile Range
NIS: National Inpatient Sample
SVT: Supraventricular Tachycardia
VF: Ventricular Fibrillation
VT: Ventricular Tachycardia.
