# Supplementary material for: Temporal trends of arrhythmias at delivery hospitalizations in the United States: Analysis from the National Inpatient Sample, 2009–2019
Source: Front Cardiovasc Med. 2022 Nov 3;9:1000298. doi: 10.3389/fcvm.2022.1000298 (PMC9668854; doi:10.3389/fcvm.2022.1000298)
Supplement: Supplementary file 1 [file Data_Sheet_1.docx]

**Supplemental Table 1.** The Reporting of studies Conducted using Observational Routinely-collected health Data (RECORD) Statement – checklist of items.

|  | **Item No.** | **RECORD items** | **Location in**  **manuscript where items are reported** |
| --- | --- | --- | --- |
| **Title and abstract** | | | |
|  | 1 | The type of data used should be specified in the title or abstract. When possible, the name of the databases used should be included.  If applicable, the geographic region and timeframe within which the study took place should be reported in the title or abstract.  If linkage between databases was conducted for the study, this should be clearly stated in the title or abstract. | Abstract |
| **Introduction** | | | |
| Background  rationale | 2 | Explain the scientific background and rationale for the investigation being reported | Page 1 |
| Objectives | 3 | State specific objectives, including any prespecified hypotheses | Page 1 |
| **Methods** | | | |
| Study Design | 4 | Present key elements of study design early in the paper | Page 3 |
| Setting | 5 | Describe the setting, locations, and relevant dates, including periods of recruitment,  exposure, follow-up, and data collection | Page 3 |
| Participants | 6 | The methods of study population selection (such as codes or algorithms used to identify subjects) should be listed in detail. If this is not possible, an explanation should be provided.  Any validation studies of the codes or algorithms used to select the population should be referenced. If validation was conducted for this study and not published elsewhere, detailed methods and results should be provided. | Page 3, Figure 1 |

|  | |  | If the study involved linkage of databases, consider use of a flow diagram or other graphical  display to demonstrate the data linkage process, including the number of individuals with linked data at each stage. |  |
| --- | --- | --- | --- | --- |
| Variables | | 7 | A complete list of codes and algorithms used to classify exposures, outcomes, confounders,  and effect modifiers should be provided. If these cannot be reported, an explanation should be provided. | Page 3,  Supplemental Table 2 and 3 |
| Data sources/ measurement | | 8 | For each variable of interest, give sources of data and details of methods of assessment (measurement).  Describe comparability of assessment methods if there is more than one group | Page 2 and 4 |
| Bias | | 9 | Describe any efforts to address potential sources of bias | Page 2 |
| Study size | | 10 | Explain how the study size was arrived at | Page 3, Figure 1 |
| Quantitative  variables | | 11 | Explain how quantitative variables were handled in the analyses. If applicable, describe  which groupings were chosen, and why | Page 4 |
| Statistical methods | | 12 | 1. Describe all statistical methods, including those used to control for confounding 2. Describe any methods used to examine subgroups and interactions 3. Explain how missing data were addressed 4. *Cohort study* - If applicable, explain how loss to follow-up was addressed   *Case-control study* - If applicable, explain how matching of cases and controls was addressed  *Cross-sectional study* - If applicable, describe analytical methods taking account of sampling strategy   1. Describe any sensitivity analyses | Page 4, 5 and 6 |
| Data access and cleaning methods | | 13 | Authors should describe the extent to which the investigators had access to the database population used to create the study population.  Authors should provide information on the data cleaning methods used in the study. | Page 3, Figure 1 |
| Linkage | | 14 | State whether the study included person-level, institutional-level, or other data linkage across two or more databases. The methods of linkage and methods of linkage quality  evaluation should be provided. | N/A |
|  | **Results** | | | |
| Participants | | 15 | Describe in detail the selection of the persons included in the study (*i.e.,* study population  selection) including filtering based on data quality, data availability and linkage. The | Page 7 |

|  |  | selection of included persons can be described in the text and/or by means of the study flow  diagram. |  |
| --- | --- | --- | --- |
| Descriptive data | 16 | 1. Give characteristics of study participants (*e.g.*, demographic, clinical, social) and information on exposures and potential confounders 2. Indicate the number of participants with missing data for each variable of interest 3. *Cohort study* - summarize follow-up time (*e.g.*, average and total amount) | Page 7, Table 1 |
| Outcome data | 17 | *Cohort study* - Report numbers of outcome events or summary measures over time  *Case-control study* - Report numbers in each exposure category, or summary measures of exposure  *Cross-sectional study* - Report numbers of outcome events or summary measures | Page 7-10, Table 2, Supplemental Table 5 |
| Main results | 18 | 1. Give unadjusted estimates and, if applicable, confounder-adjusted estimates and their precision (e.g., 95% confidence interval). Make clear which confounders were adjusted for and why they were included 2. Report category boundaries when continuous variables were categorized 3. If relevant, consider translating estimates of relative risk into absolute risk for a meaningful time period | Page 7-10, Table 2, Supplemental Table 5 |
| Other analyses | 19 | Report other analyses done—e.g., analyses of subgroups and interactions, and sensitivity analyses | Page 10,  Supplemental Table 6 and 7 |
| **Discussion** | | | |
| Key results | 20 | Summarize key results with reference to study objectives | Page 11 |
| Limitations | 21 | Discuss the implications of using data that were not created or collected to answer the specific research question(s). Include discussion of misclassification bias, unmeasured confounding, missing data, and changing eligibility over time, as they pertain to the study  being reported. | Page 15 |
| Interpretation | 22 | Give a cautious overall interpretation of results considering objectives, limitations,  multiplicity of analyses, results from similar studies, and other relevant evidence | Page 15 |
| Generalizability | 23 | Discuss the generalizability (external validity) of the study results | Page 15 |
| **Other Information** | | | |
| Funding | 24 | Give the source of funding and the role of the funders for the present study and, if  applicable, for the original study on which the present article is based | Page 18 |
| Accessibility of protocol, raw data, and  programming code | 25 | Authors should provide information on how to access any supplemental information such as the study protocol, raw data, or programming code. | Page 2 |

*Reference: Benchimol EI, Smeeth L, Guttmann A, Harron K, Moher D, Petersen I, Sørensen HT, von Elm E, Langan SM, the RECORD Working Committee. The Reporting of studies Conducted using Observational Routinely-collected health Data (RECORD) Statement. *PLoS Medicine* 2015.

**Supplemental Table 2. International Classification of Diseases, Tenth Revision, Clinical Modification (ICD-9 &10-CM) codes used to identify hospitalization diagnosis (Delivery) and clinical covariates in hospitalizations with delivery.**

| **Variable Name** | **ICD-10-CM codes** | **ICD-9-CM codes** |
| --- | --- | --- |
| ***Dx*** |  |  |
| **Delivery**  (Diagnosis code) | O60, O61, O62, O63, O64, O65, O66, O67, O68, O69, O70, O71, O72, O73, O74, O75, O76, O77, O80, Z37, Z38 | 650, 651, 652, 653,  654, 655, 656, 657, 658, 659, 660, 662, 663, 664, 665, 666, 667, 668, 669, V27 |
| Procedure code | 10D0, 10E0 | 72, 73, 75 |
| **C Section**  Diagnosis | O82 | 669.7 |
| Procedure code | 10D00Z0, 10D00Z1,  10D00Z2 | 74 |
| ***Co-morbidities*** |  |  |
| Smoking | F17 | V15.82, 305.1 |
| Hyperlipidemia | E78 | 272.4 |
| OSA | G47.33 | 327.23 |
| Prior MI | I25.2 | 412 |
| Cardiac Arrhythmias | I48.0, I48.1, I48.2, I48.91, I48.3, I48.4, I48.92, I47.1, I47.2, I49.01 | 427.31, 427.32, 427.0,  427.1, 427.41 |
|  |  |  |
| Atrial Fibrillation | I48.0, I48.1, I48.2, I48.91 | 427.31 |
| Atrial Flutter | I48.3, I48.4, I48.92 | 427.32 |
| Supraventricular Tachycardia | I47.1 | 427.0 |
| Ventricular tachycardia | I47.2 | 427.1 |
| Ventricular Fibrillation | I49.01 | 427.41 |

**Supplemental Table 3: The International Classification of Diseases, Tenth Revision, Clinical Modification (ICD-9/10-CM) codes used for study outcomes**

| **Variable** | **ICD-10-CM codes** | **ICD-9-CM codes** |
| --- | --- | --- |
| Cardiogenic Shock | R57.0 | 785.51 |
| Cardiac Arrest | I46 | 427.5 |
| Acute Heart Failure | I50.21, I50.31, I50.41, I50.23, I50.33, I50.43, I50.811, I50.813 | 428.21, 428.23, 428.31, 428.33, 428.41, 428.43 |
| Ischemic Stroke | I63, I67.81, I67.82 | 436, 433.01, 433.11, 433.21, 433.31, 433.81, 433.91, 434.01, 434.11, 434.91 |
| Hemorrhagic stroke | I60, I61, I62 | 430, 431, 432 |
| Preterm | P073 | 644 |
| Preeclampsia | O14 | 642 |
| Gestational DM | O244 | 648.0 |
| Placental Abruption | O45 | 641.2 |
| Placenta previa | O44 | 641.1, 641.0 |
| Fetal Death | O36.4 | 656.4, 768.0 |
| Post-partum cardiomyopathy | O90.3 | 674.52, 674.54 |
| Maternal Shock | O75.1 | 669.11, 669.12, 669.14 |

**Supplemental Table 4.** Methodological checklist for studies published using the National Inpatient Sample survey data.

| **Section A. Research Design** | |
| --- | --- |
| Yes | **Does the study consider that it can only detect diseases conditions, procedures, and diagnostic tests in hospital settings?** |
| Yes | **Does the study acknowledge that it includes encounters, not individual patients?** |
| Yes | **Does the study avoid diagnosis/procedure-specific volume assessment for units that are not a part of the sampling frame of the NIS, and therefore not representatively sampled, including**   1. **Geographic units, like U.S. states** 2. **Healthcare facilities (after 2011)** 3. **Individual healthcare providers?** |
| **Section B. Data Interpretation** | |
| Yes | **Does the study attempt to identify disease conditions or procedures of interest using administrative codes or their combinations that have been previously validated?** |
| Yes | **Does the study limit its assessment to only in-hospital outcomes, rather than those occurring after discharge?** |
| Yes | **Does the study distinguish complications from comorbidities or clearly not where it cannot?** |
| **Section C: Data Analysis** | |
| Yes | **Does the study clearly account for the survey design of the NIS and its components—clustering, stratification, and weighting?** |
| Yes | **Does the study adequately address changes in data structure over time (for trend analyses)?** |

*Reference: Khera R, Krumholz HM. With Great Power Comes Great Responsibility. Circulation: Cardiovascular Quality and Outcomes. 2017;10(7):e003846.
